# 4,6,7,9,10,12,13,15,16,18-Decahydro-1,3-dithiolo[4,5-*l*][1,4,7,10,15]trioxadithia­cyclo­hepta­decine-2-thione

**DOI:** 10.1107/S1600536810016946

**Published:** 2010-05-15

**Authors:** Rui-Bin Hou, Bao Li, Bing-Zhu Yin, Li-Xin Wu

**Affiliations:** a Key Laboratory of Organism Functional Factors of the Changbai Moutain, Yanbian University, Ministry of Education, Yanji 133002, People’s Republic of China; b State Key Laboratory of Supramolecular Structure and Materials, College of Chemistry, Jilin University, Changchun 130012, People’s Republic of China

## Abstract

The title compound, C_13_H_20_O_3_S_5_, is bis­ected by a crystallographic twofold rotation axis, which relates the two halves of the mol­ecule to one another: one S, one C and one O atom lie on the axis. The thione S atom lies in the plane of the five-membered rings with an r.m.s. deviation of 0.0042 (5) Å. Parts of the 17-membered macrocycle were refined using a two-part disorder model [occupancies of  0.553 (14) and  0.447 (14)]. There are no noteworthy inter­molecular inter­actions.

## Related literature

Thia­crown ether annulated 1,3-dithiol-2-thione is a key inter­mediate of the crown ether-bearing redox-active tetra­thia­fulvalene moiety, see: Moore *et al.* (2000 ▶). For details of the synthesis, see: Chen *et al.* (2005 ▶). For a related structure, see: Hou *et al.* (2009 ▶)
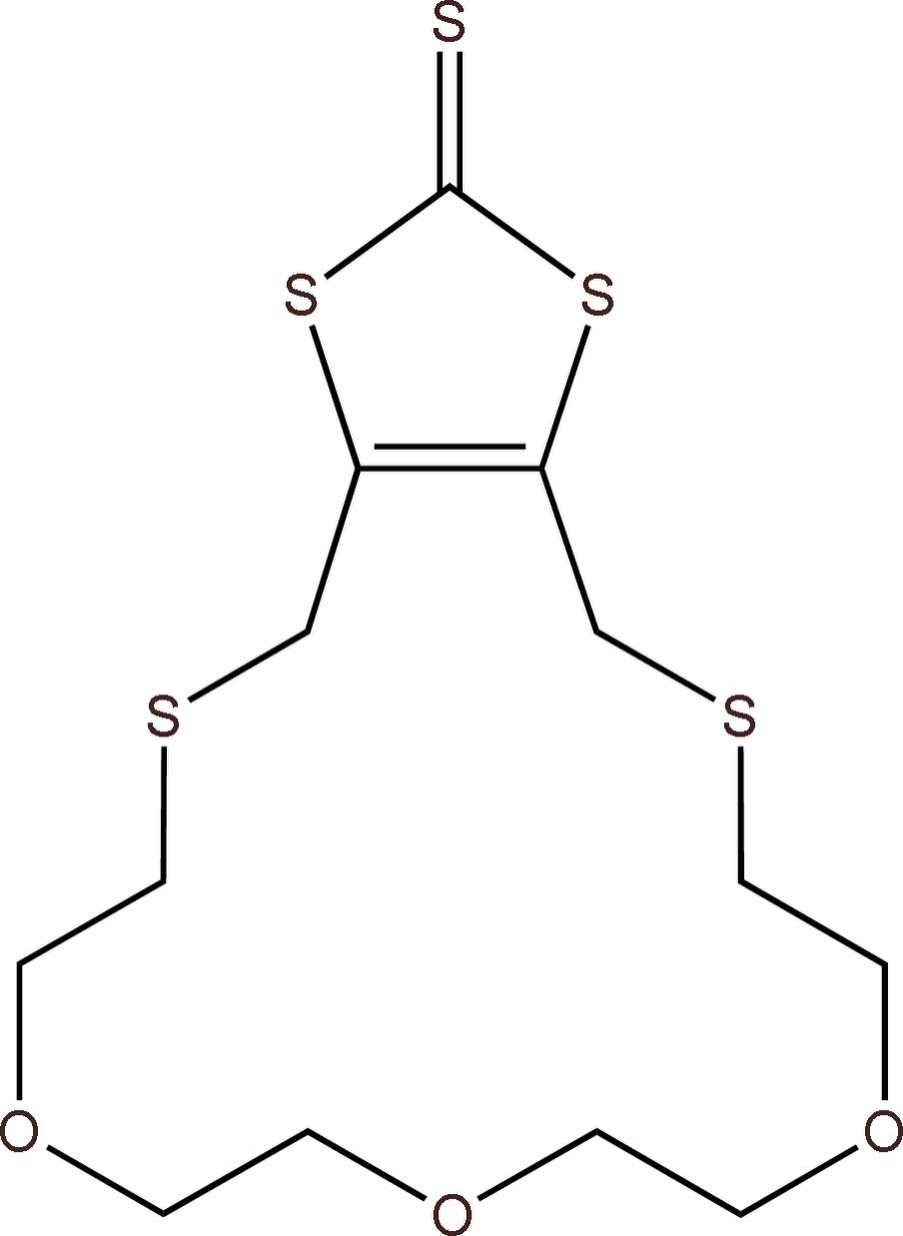


## Experimental

### 

#### Crystal data

C_13_H_20_O_3_S_5_
*M* *_r_* = 384.59
Monoclinic, 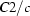

*a* = 14.040 (3) Å
*b* = 13.616 (3) Å
*c* = 10.004 (2) Å
β = 110.89 (3)°
*V* = 1786.6 (6) Å^3^
*Z* = 4
Mo *K*α radiation
μ = 0.65 mm^−1^
*T* = 290 K
0.13 × 0.13 × 0.12 mm

#### Data collection

Rigaku R-AXIS RAPID diffractometer
Absorption correction: multi-scan (*ABSCOR*; Higashi, 1995 ▶) *T*_min_ = 0.920, *T*_max_ = 0.926
8683 measured reflections
2054 independent reflections
1795 reflections with *I* > 2σ(*I*)
*R*_int_ = 0.022

#### Refinement

*R*[*F* ^2^ > 2σ(*F* ^2^)] = 0.039
*wR*(*F* ^2^) = 0.103
*S* = 1.05
2054 reflections
116 parameters
31 restraints
H-atom parameters constrained
Δρ_max_ = 0.38 e Å^−3^
Δρ_min_ = −0.40 e Å^−3^

### 

Data collection: *RAPID-AUTO* (Rigaku, 1998 ▶); cell refinement: *RAPID-AUTO*; data reduction: *CrystalStructure* (Rigaku/MSC, 2002 ▶); program(s) used to solve structure: *SHELXS97* (Sheldrick, 2008 ▶); program(s) used to refine structure: *SHELXL97* (Sheldrick, 2008 ▶); molecular graphics: *PLATON* (Spek, 2009 ▶); software used to prepare material for publication: *SHELXL97*.

## Supplementary Material

Crystal structure: contains datablocks global, I. DOI: 10.1107/S1600536810016946/nk2030sup1.cif

Structure factors: contains datablocks I. DOI: 10.1107/S1600536810016946/nk2030Isup2.hkl

Additional supplementary materials:  crystallographic information; 3D view; checkCIF report

## Figures and Tables

**Fig. 1.**
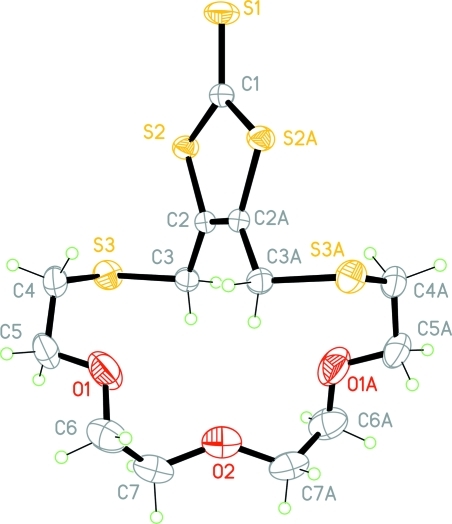
The asymmetric of title compound, with the atom numbering. The disordered C6', C7' and their attached H atoms are omitted for clarity. Displacement ellipsoids of non-H atoms are drawn at the 30% probalility level.

## supplementary crystallographic information

### Comment

Thiacrown ether annulated 1,3-dithiol-2-thione has been intensively investigated because it is a key intermediate of the crown ether bearing redox-active tetrathiafulvalene moiety (Moore *et al.*, 2000). Herein, we report the crystal structure of the title compound, (I).

The molecule structure of title compound, C_13_H_20_O_3_S_5_, is shown in Fig. 1. All bond lengths and angles are unexceptional and comparable with the related structure (Hou *et al.*, 2009). The C6, C7 and C6', C7' atoms were refined using a two-part disorder model with a major:minor occupancy ratio of 55:45.

### Experimental

The title compound was prepared according to the literature (Chen *et al.*, 2005). Single crystals suitable for X-ray diffraction were prepared by slow evaperation a mixture of dichloromethane and petroleum (60-90 °C) at room temperature.

### Refinement

C-bound H-atoms were placed in calculated positions (C—H 0.97 Å) and were included in the refinement with U_iso_(H) = 1.2 U_eq_(C).  Atoms C6, C7 and C6', C7'  were refined using a two-part disorder model with a major:minor occupancy ratio of 55:45. Mild rigid bond restraints were used on the disordered components.

### Crystal data

**Table:**

C_13_H_20_O_3_S_5_	*F*(000) = 808
*M**_r_* = 384.59	*D*_x_ = 1.430 Mg m^−^^3^
Monoclinic, *C*2/*c*	Mo *K*α radiation, λ = 0.71073 Å
Hall symbol: -C 2yc	Cell parameters from 7120 reflections
*a* = 14.040 (3) Å	θ = 3.0–27.5°
*b* = 13.616 (3) Å	µ = 0.65 mm^−^^1^
*c* = 10.004 (2) Å	*T* = 290 K
β = 110.89 (3)°	Block, yellow
*V* = 1786.6 (6) Å^3^	0.13 × 0.13 × 0.12 mm
*Z* = 4	

### Data collection

**Table:**

Rigaku R-AXIS RAPID diffractometer	2054 independent reflections
Radiation source: fine-focus sealed tube	1795 reflections with *I* > 2σ(*I*)
graphite	*R*_int_ = 0.022
ω scans	θ_max_ = 27.5°, θ_min_ = 3.0°
Absorption correction: multi-scan (*ABSCOR*; Higashi, 1995)	*h* = −18→17
*T*_min_ = 0.920, *T*_max_ = 0.926	*k* = −15→17
8683 measured reflections	*l* = −12→12

### Refinement

**Table:**

Refinement on *F*^2^	Primary atom site location: structure-invariant direct methods
Least-squares matrix: full	Secondary atom site location: difference Fourier map
*R*[*F*^2^ > 2σ(*F*^2^)] = 0.039	Hydrogen site location: inferred from neighbouring sites
*wR*(*F*^2^) = 0.103	H-atom parameters constrained
*S* = 1.05	*w* = 1/[σ^2^(*F*_o_^2^) + (0.0458*P*)^2^ + 1.730*P*] where *P* = (*F*_o_^2^ + 2*F*_c_^2^)/3
2054 reflections	(Δ/σ)_max_ < 0.001
116 parameters	Δρ_max_ = 0.38 e Å^−^^3^
31 restraints	Δρ_min_ = −0.40 e Å^−^^3^

### Special details

**Table:**

Experimental. (See detailed section in the paper)
Geometry. All esds (except the esd in the dihedral angle between two l.s. planes) are estimated using the full covariance matrix. The cell esds are taken into account individually in the estimation of esds in distances, angles and torsion angles; correlations between esds in cell parameters are only used when they are defined by crystal symmetry. An approximate (isotropic) treatment of cell esds is used for estimating esds involving l.s. planes.
Refinement. Refinement of *F*^2^ against ALL reflections. The weighted *R*-factor wR and goodness of fit *S* are based on *F*^2^, conventional *R*-factors *R* are based on *F*, with *F* set to zero for negative *F*^2^. The threshold expression of *F*^2^ > σ(*F*^2^) is used only for calculating *R*-factors(gt) etc. and is not relevant to the choice of reflections for refinement. *R*-factors based on *F*^2^ are statistically about twice as large as those based on *F*, and *R*- factors based on ALL data will be even larger.

### Fractional atomic coordinates and isotropic or equivalent isotropic displacement parameters (Å^2^)

**Table:**

	*x*	*y*	*z*	*U*_iso_*/*U*_eq_	Occ. (<1)
S1	0.0000	−0.12281 (6)	0.2500	0.0692 (3)	
S2	0.05129 (4)	0.06857 (4)	0.14909 (6)	0.04829 (17)	
S3	0.17462 (5)	0.26384 (5)	0.11969 (8)	0.0693 (2)	
O1	0.17749 (17)	0.40044 (19)	0.3861 (3)	0.1067 (8)	
O2	0.0000	0.5091 (3)	0.2500	0.1381 (16)	
C1	0.0000	−0.0016 (2)	0.2500	0.0456 (6)	
C2	0.02260 (13)	0.18371 (13)	0.2012 (2)	0.0390 (4)	
C3	0.04873 (15)	0.27043 (15)	0.1292 (2)	0.0493 (5)	
H3A	0.0440	0.3295	0.1807	0.059*	
H3B	−0.0010	0.2757	0.0330	0.059*	
C4	0.25511 (19)	0.2730 (2)	0.3037 (4)	0.0816 (9)	
H4A	0.2278	0.2308	0.3594	0.098*	
H4B	0.3221	0.2481	0.3138	0.098*	
C5	0.2672 (2)	0.3743 (2)	0.3658 (4)	0.0896 (10)	
H5A	0.3238	0.3758	0.4564	0.108*	
H5B	0.2813	0.4205	0.3013	0.108*	
C6	0.1737 (6)	0.4901 (6)	0.4396 (11)	0.092 (2)	0.553 (14)
H6A	0.2409	0.5199	0.4753	0.111*	0.553 (14)
H6B	0.1473	0.4868	0.5169	0.111*	0.553 (14)
C7	0.1044 (10)	0.5461 (6)	0.3178 (12)	0.099 (3)	0.553 (14)
H7A	0.1004	0.6126	0.3501	0.118*	0.553 (14)
H7B	0.1351	0.5497	0.2451	0.118*	0.553 (14)
C6'	0.1649 (8)	0.5194 (7)	0.3562 (16)	0.083 (3)	0.447 (14)
H6'1	0.2130	0.5549	0.4360	0.100*	0.447 (14)
H6'2	0.1785	0.5356	0.2703	0.100*	0.447 (14)
C7'	0.0574 (11)	0.5476 (8)	0.3385 (11)	0.092 (3)	0.447 (14)
H7'1	0.0459	0.5333	0.4266	0.110*	0.447 (14)
H7'2	0.0491	0.6178	0.3219	0.110*	0.447 (14)

### Atomic displacement parameters (Å^2^)

**Table:**

	*U*^11^	*U*^22^	*U*^33^	*U*^12^	*U*^13^	*U*^23^
S1	0.0894 (6)	0.0347 (4)	0.0982 (7)	0.000	0.0513 (5)	0.000
S2	0.0524 (3)	0.0401 (3)	0.0612 (3)	0.00140 (19)	0.0310 (2)	−0.0020 (2)
S3	0.0700 (4)	0.0671 (4)	0.0918 (5)	−0.0035 (3)	0.0543 (4)	0.0127 (3)
O1	0.0793 (14)	0.1025 (17)	0.142 (2)	−0.0370 (13)	0.0438 (14)	−0.0511 (16)
O2	0.110 (3)	0.067 (2)	0.225 (5)	0.000	0.044 (3)	0.000
C1	0.0432 (13)	0.0374 (13)	0.0576 (15)	0.000	0.0199 (12)	0.000
C2	0.0321 (8)	0.0354 (9)	0.0478 (9)	−0.0004 (6)	0.0123 (7)	0.0006 (7)
C3	0.0459 (10)	0.0444 (10)	0.0561 (11)	−0.0011 (8)	0.0165 (9)	0.0090 (9)
C4	0.0423 (12)	0.0699 (17)	0.122 (2)	−0.0026 (11)	0.0168 (14)	0.0213 (16)
C5	0.0518 (15)	0.086 (2)	0.115 (2)	−0.0222 (13)	0.0109 (15)	0.0036 (18)
C6	0.109 (5)	0.077 (4)	0.080 (5)	−0.023 (3)	0.021 (3)	−0.014 (3)
C7	0.112 (5)	0.054 (3)	0.126 (5)	−0.018 (4)	0.038 (5)	−0.010 (3)
C6'	0.092 (5)	0.063 (5)	0.100 (7)	−0.029 (4)	0.043 (5)	−0.029 (5)
C7'	0.108 (7)	0.081 (5)	0.094 (5)	−0.003 (5)	0.045 (5)	−0.037 (4)

### Geometric parameters (Å, °)

**Table:**

S1—C1	1.650 (3)	C3—H3B	0.9700
S2—C1	1.7231 (16)	C4—C5	1.497 (4)
S2—C2	1.7440 (18)	C4—H4A	0.9700
S3—C4	1.788 (3)	C4—H4B	0.9700
S3—C3	1.806 (2)	C5—H5A	0.9700
O1—C6	1.342 (7)	C5—H5B	0.9700
O1—C5	1.391 (4)	C6—C7	1.472 (14)
O1—C6'	1.645 (12)	C6—H6A	0.9700
O2—C7'	1.095 (11)	C6—H6B	0.9700
O2—C7'^i^	1.095 (11)	C7—H7A	0.9700
O2—C7	1.467 (11)	C7—H7B	0.9700
O2—C7^i^	1.467 (11)	C6'—C7'	1.506 (15)
C1—S2^i^	1.7231 (16)	C6'—H6'1	0.9700
C2—C2^i^	1.340 (4)	C6'—H6'2	0.9700
C2—C3	1.495 (3)	C7'—H7'1	0.9700
C3—H3A	0.9700	C7'—H7'2	0.9700
			
C1—S2—C2	97.69 (10)	O1—C5—H5B	109.9
C4—S3—C3	102.32 (13)	C4—C5—H5B	109.9
C6—O1—C5	117.2 (4)	H5A—C5—H5B	108.3
C6—O1—C6'	32.8 (4)	O1—C6—C7	104.4 (8)
C5—O1—C6'	105.6 (4)	O1—C6—H6A	110.9
C7'—O2—C7'^i^	122.7 (13)	C7—C6—H6A	110.9
C7'—O2—C7	30.3 (5)	O1—C6—H6B	110.9
C7'^i^—O2—C7	122.3 (7)	C7—C6—H6B	110.9
C7'—O2—C7^i^	122.3 (7)	H6A—C6—H6B	108.9
C7'^i^—O2—C7^i^	30.3 (5)	O2—C7—C6	117.5 (8)
C7—O2—C7^i^	139.8 (8)	O2—C7—H7A	107.9
S1—C1—S2^i^	123.68 (8)	C6—C7—H7A	107.9
S1—C1—S2	123.68 (8)	O2—C7—H7B	107.9
S2^i^—C1—S2	112.65 (15)	C6—C7—H7B	107.9
C2^i^—C2—C3	127.72 (11)	H7A—C7—H7B	107.2
C2^i^—C2—S2	115.95 (6)	C7'—C6'—O1	108.1 (8)
C3—C2—S2	116.30 (14)	C7'—C6'—H6'1	110.1
C2—C3—S3	113.52 (14)	O1—C6'—H6'1	110.1
C2—C3—H3A	108.9	C7'—C6'—H6'2	110.1
S3—C3—H3A	108.9	O1—C6'—H6'2	110.1
C2—C3—H3B	108.9	H6'1—C6'—H6'2	108.4
S3—C3—H3B	108.9	O2—C7'—C6'	113.0 (9)
H3A—C3—H3B	107.7	O2—C7'—C7'^i^	28.7 (6)
C5—C4—S3	115.3 (2)	C6'—C7'—C7'^i^	125.7 (13)
C5—C4—H4A	108.4	O2—C7'—H7'1	109.0
S3—C4—H4A	108.4	C6'—C7'—H7'1	109.0
C5—C4—H4B	108.4	C7'^i^—C7'—H7'1	118.7
S3—C4—H4B	108.4	O2—C7'—H7'2	109.0
H4A—C4—H4B	107.5	C6'—C7'—H7'2	109.0
O1—C5—C4	108.8 (2)	C7'^i^—C7'—H7'2	80.4
O1—C5—H5A	109.9	H7'1—C7'—H7'2	107.8
C4—C5—H5A	109.9		

Symmetry codes: (i) −*x*, *y*, −*z*+1/2.
